# The effects of propolis supplementation on metabolic parameters: A systematic review and meta-analysis of randomized controlled clinical trials

**DOI:** 10.22038/AJP.2021.18046

**Published:** 2021

**Authors:** Alireza Gheflati, Zahra Dehnavi, Aliyeh Ghannadzadeh Yazdi, Zahra Khorasanchi, Hamidreza Raeisi-Dehkordi, Golnaz Ranjbar

**Affiliations:** 1 *Department of Nutrition, School of Medicine, Mashhad University of Medical Sciences, Mashhad, Iran*; 2 *Department of Nutrition, School of Public Health, Shahid Sadoughi University of Medical Sciences, Yazd, Iran*

**Keywords:** Propolis, Lipid profile, Liver enzyme, Metabolic parameter

## Abstract

**Objective:**

Propolis is a sticky, resinous substance produced by honeybees from various plants. Various biological properties of propolis and its extracts have been recognized in previous studies including the antiseptic, anti-inflammatory, antioxidant, antiviral, hepatoprotective, antitumor, antibacterial and antimycotic properties. This study aimed to summarize the effect of propolis on metabolic parameters in human adults using systematic review and meta-analysis.

**Materials and Methods:**

A comprehensive systematic search was performed in ISI Web of Science, PubMed, Scopus, and Google Scholar up to July 2020 for controlled clinical trials evaluating the impact of propolis on lipid profile and liver enzyme biomarkers. A random effects model was used to calculate the weighted mean difference (WMD) and 95% confidence interval (CI) as the difference between the mean for the intervention and control groups.

**Results:**

The present meta-analysis included six randomized controlled trials. There was significant reduction in Aspartate Aminotransferase (AST) in comparison to the control groups (WMD=-2.01; 95% CI: -3.93--0.10; p=0.039). However, a non-significant effect was observed in Triglycerides (TG), Total cholesterol (TC), low-density lipoprotein (LDL), High-density lipoprotein (HDL) (WMD=-0.05 mg/dl; 95% CI: -0.27-0.18; p=0.688; WMD=7.08 mg/dl; 95% CI: -37.31-51.46; p=0.755; WMD=-0.94 mg/dl; 95% CI: -6.64-4.77; p=0.747; WMD=3.14 mg/dl; 95% CI: -1.84-8.13; p=0.216, respectively).

**Conclusion:**

Current meta-analysis revealed that propolis supplementation can reduce AST; nevertheless, there was no significant effect on lipid profile indices and ALT.

## Introduction

Propolis is a sticky, resinous substance produced by honeybees from different plants. The term 'propolis' is a Greek word, in which 'pro' means defense, and 'polis' means city/community or the beehive (Castaldo and Capasso, 2002 ▶). Propolis is one of the few natural drugs that is usually used as a dietary supplement for human health in traditional medicine (Lisičić et al., 2014 ▶; Kocot et al., 2018 ▶).

Various biological properties of propolis and its extracts have been recognized in previous studies, including the antiseptic, anti-inflammatory, antioxidant, cytotoxic, antiviral, hepatoprotective, antitumor, antibacterial, antimycotic, antifungal, antiulcer, anticancer, and immune function-stimulating properties (Bankova et al., 2000 ▶; Toreti et al., 2013 ▶; Pasupuleti et al., 2017 ▶). More than 300 compound have been identified in propolis, and almost all the biological activities of this substance are closely related to the presence of phenolic components such as flavonoids, terpenes, aromatic aldehydes, beta-steroids, and alcohols (Mani et al., 2006 ▶; Viuda‐Martos et al., 2008 ▶).

Cardiometabolic disorders such as the hypertension, metabolic syndrome, diabetes mellitus, dyslipidemia, fatty liver, and cardiovascular diseases have severe consequences and they are related to a higher risk of mortality and morbidity and high social costs worldwide (Guo et al., 2014 ▶). Documented evidence attests to the positive effects of nutritional compounds with anti-inflammatory and antioxidant features on the prevention and management of cardiometabolic disorders (Rocha et al., 2014 ▶; Soory, 2012 ▶). As such, there has been growing notice in the utilization of these antioxidants or anti-inflammatory compounds for prevention and treatment of cardiometabolic disorders. Furthermore, emerging evidence suggests that propolis as an antioxidant compound could improve various cardiometabolic risk factors, which makes it the most suitable candidate for the treatment of cardiometabolic disorders (Mujica et al., 2017 ▶; Afsharpour et al., 2019 ▶; Pasupuleti et al., 2017 ▶).

Previous findings have indicated the helpful effects of propolis on oxidative stress and antioxidant status through enhancing glutathione and decreasing malondialdehyde and thiobarbituric acid reactive substances as oxidative stress markers in humans (Mujica et al., 2017 ▶; Jasprica et al., 2007 ▶; Gao et al., 2018 ▶). As well, the therapeutic role of propolis in the prevention and treatment of diabetes mellitus (DM) has been approved in several clinical studies (Afsharpour et al., 2019 ▶; Hesami et al., 2019 ▶). A systematic review and meta-analysis revealed that propolis supplementation had beneficial impacts on the control of the glycemic status in patients with type II DM (Karimian et al., 2019 ▶), while numerous studies have demonstrated the beneficial impacts of propolis on inflammatory biomarkers, such as tumour necrosis factor α (TNF-α ) and C-reactive protein (CRP). In addition, a recent systematic review and meta-analysis showed the potential impacts of propolis on the improvement of serum CRP and TNF-α levels (Jalali et al., 2020 ▶). 

Incompatible findings have been proposed about the effects of propolis on the lipid profile and liver biomarkers. For instance, a clinical study revealed that propolis has positive effects on the improvement of high-density lipoprotein cholesterol (HDL-c) and reduced risk of cardiovascular diseases. However, no beneficial effects were attributed to propolis for other serum lipids and liver biomarkers like alanine aminotransferase (ALT) and aspartate aminotransferase (AST) in the mentioned study (Mujica et al., 2017 ▶). In another study, Zakerkish et al. reported that the AST and ALT levels significantly decreased after 90 days of propolis supplementation, while these biomarkers had no significant changes compared to the placebo group (Zakerkish et al., 2019 ▶).

Considering that the evidence regarding the impact of propolis on lipid profile and liver biomarkers as significant biomarkers in cardiometabolic disorders, has not been substantiated, this systematic review and meta-analysis aimed to provide an accurate evaluation of the effects of propolis on lipid profile and liver biomarkers. 

## Materials and Methods

A comprehensive and systematic search was designed in accordance with the preferred reporting items for systematic reviews and meta-analyses (PRISMA) guidelines (Moher et al., 2015 ▶). The protocol of the present study was registered in the International Prospective Register of Systematic Reviews (PROSPERO) database (http://www.crd.york.ac.uk/PROSPERO; registration No. CRD42020191750). 

**Search strategy**

A comprehensive and systematic search was performed in databases such as PubMed, Scopus, ISI, Web of Science, and Google Scholar until July 2020 without language and time restriction using MeSH and non-MeSH query, including ("green propolis" OR "red propolis" OR propolis OR "bee glue" OR "bee bread OR "bee propolis" OR propolis* OR "propolis extract*" OR "brown propolis" OR "honey bee propolis" OR propolisina) AND (intervention OR Intervention* OR trial OR randomized OR randomised OR random OR randomly OR placebo OR assignment OR "clinical trial" OR RCT OR "clinical trials as topic" OR cross-over OR parallel).

The titles and abstracts of the relevant articles were independently screened by two reviewers (A. G. Y. and Z. D.) to eliminate the articles that were clearly irrelevant, and differences were concluded through discourse with the other reviewers (Z. K. H. and A. G.). Additional relevant studies were also retrieved by screening the reference lists of the related articles manually.

**Eligibility criteria**

The eligibility criteria for the articles were as listed below: 1) original randomized controlled trials (RCTs); 2) evaluation of the effects of propolis on humans; 3) participants aged ≥18 years and 4) reported effects of any forms of propolis supplementation/extracts on triglycerides, HDL, low-density lipoprotein (LDL), total cholesterol (TC), AST, and ALT. The exclusion criteria were as follows: 1) assessment of the acute effects of propolis supplementation; 2) *in-vitro*, animal or review studies; 3) no investigation of lipid profile indicators or liver enzyme and 4) evaluation of a propolis supplementation in combination with other components not comparable to a control group. 

**Data extraction**

After selection of the eligible articles, the following data were extracted: the first author's name, publication year, location of the study, number of the subjects, age and gender of the subjects, study design, type and dose of propolis and placebo intake, and duration of the study. In addition, the mean and standard deviation (SD) of the outcome data at baseline and after the follow-up period or their change values were extracted. If the SD value was not available, it would be calculated using the following formula: SD=SEM×√n where n represents the number of the subjects per each group. 

Data extraction was executed independently by three reviewers (A. G., Z. K. H., and Z. D.), and the process was double-checked by the other authors (A. G. and H. R. D.). Group consultation resolved the disagreements between the reviewers. 

**Risk of bias**

The quality of the eligible studies was evaluated by the Cochrane Collaboration's tool for the systematic review of interventions (Higgins et al., 2019 ▶) considering six items, including the adequacy of sequence generation, allocation concealment, blinding of the participants, personnel and outcome assessment, incomplete outcome data, selective outcome reporting, and other potential sources of bias. The selected studies were stratified as Yes (low risk of bias), No (high risk of bias), and Unclear (uncertain risk of bias). The quality of the articles was graded as weak, fair or good if the *<*3, 3, and *≥*4 domains were rated as low-risk, respectively.

**Statistical analysis**

The mean change values and SDs were extracted on TG, LDL, HDL, TC, AST, and ALT for propolis supplementation and control groups/period to calculate the difference in means and their standard errors (SEs) for use as the effect size for the meta-analysis. In addition, Hedges’g analysis would be used as the effect size to conduct the meta-analysis if favorable values reported the use of various units, and they could not be converted into a single unit. If the change values were not reported, the correlation-coefficient would be considered 0.5 for the baseline and follow-up data to estimate the mean changes and their SDs. To ensure that the meta-analysis was not sensitive to the selected correlation-coefficient, all the analyses were replicated at the correlation-coefficients of 0.2 and 0.8. 

To calculate the weighted mean difference (WMD), the random effects model was used considering the inter‐study heterogeneity, and the corresponding 95% confidence intervals (CIs) were considered the summary estimate. By Cochran's Q-test and I^2^ statistic, the heterogeneity between the studies was evaluated. The potential publication bias was also evaluated using the visual inception of the funnel plots and Egger and Begg regression test. Data analysis was performed in STATA version 11.2 (Stata Corp, College Station, TX), and statistically significant effects were defined with the two‐tailed p-value of less than 0.05.

## Results

**Selected studies**

**Figure 1:**
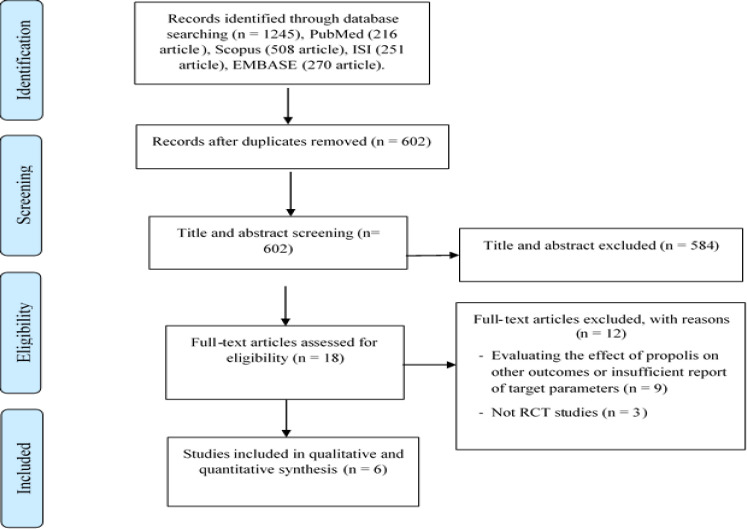
Flow diagram of study selection process

The preliminary database search resulted in the identification of 1245 potentially relevant articles. After the elimination of duplicate references, 602 articles remained for the screening of the titles and abstracts, out of which, 18 full-text articles were further assessed. After the meticulous reading of the selected full-text articles, 12 articles were subsequently eliminated from the systematic review (Figure 1). Finally, six eligible RCTs were included in this systematic review and meta-analysis, four of which reported the effects of propolis supplementation on TG (Fukuda et al., 2015 ▶; Mujica et al., 2017 ▶; Samadi et al., 2017 ▶; Zakerkish et al., 2019 ▶), four reported the effects on TC (Fukuda et al., 2015 ▶; Mujica et al., 2017 ▶; Samadi et al., 2017 ▶; Zakerkish et al., 2019 ▶), four reported the effects on HDL (Fukuda et al., 2015 ▶; Mujica et al., 2017 ▶; Samadi et al., 2017 ▶; Zakerkish et al., 2019 ▶), four reported the effects on LDL (Fukuda et al., 2015 ▶; Mujica et al., 2017 ▶; Samadi et al., 2017 ▶; Zakerkish et al., 2019 ▶), four reported the effects on AST (Afsharpour et al., 2017 ▶; Mujica et al., 2017 ▶; Silveira et al., 2019 ▶; Zakerkish et al., 2019 ▶), and four reported the effects on ALT (Afsharpour et al., 2017 ▶; Mujica et al., 2017 ▶; Silveira et al., 2019 ▶; Zakerkish et al., 2019 ▶).

**Characteristics of the selected studies**

Table 1 shows the main characteristics of the six eligible RCTs. The reviewed studies were published during 2015-2019, three of which were performed in Iran (Afsharpour et al., 2017 ▶; Samadi et al., 2017 ▶; Zakerkish et al., 2019 ▶), one was conducted in Brazil (Silveira et al., 2019 ▶), one was performed in Chile (Mujica et al., 2017 ▶), and one was done in Japan (Fukuda et al., 2015 ▶). The duration of the follow-up period of the studies was 56-365 days. In total, 417 participants were randomly designated to these investigation, and 93.5% (n=390) finished the trials. The mean age of the participants was within the range of 44.5-63.7 years. Notably, all the reviewed studies were performed on males and females (Afsharpour et al., 2017 ▶; Fukuda et al., 2015 ▶; Mujica et al., 2017 ▶; Samadi et al., 2017 ▶; Silveira et al., 2019 ▶; Zakerkish et al., 2019 ▶). In most of the studies, propolis was used in the shape of capsules/tablets/pills (Afsharpour et al., 2017 ▶; Fukuda et al., 2015 ▶; Samadi et al., 2017 ▶; Silveira et al., 2019 ▶; Zakerkish et al., 2019 ▶), while in only one study, propolis was used in the form of drops (Afsharpour et al., 2017 ▶; Fukuda et al., 2015 ▶; Samadi et al., 2017 ▶; Silveira et al., 2019 ▶; Zakerkish et al., 2019 ▶). In addition, the participants had variable baseline conditions, including type II DM (Afsharpour et al., 2017 ▶; Fukuda et al., 2015 ▶; Samadi et al., 2017 ▶; Zakerkish et al., 2019 ▶) and chronic kidney disease (CKD) (Silveira et al., 2019 ▶).

**Risk of bias**

Table 2 shows the details of the methodological quality assessment. Five out of the six reviewed studies explained the randomization method of the subjects, such as stratified randomization (Silveira et al., 2019), table of random numbers (Afsharpour et al., 2017 ▶; Samadi et al., 2017 ▶), Microsoft excel spreadsheet (Mujica et al., 2017 ▶), and software (Zakerkish et al., 2019 ▶), and in only one trial, there was no mention of the randomization procedure (Fukuda et al., 2015 ▶). Moreover, only one study defined the precise method of allocation concealment (Fukuda et al., 2015 ▶). Manifestation of bias due to selective reporting or attrition was not detected in the studies.

**Table 1:** Characteristics of the randomized clinical trials included in the present systematic review and meta-analysis

Side effect	Notes on participants	Diet type	Reported data	Comparison group	Intervention group	Duration (days)	Study design	Mean age of subjects (year)	Country	Number and gender of subjects (F/M)	First author (publication year)
None	CKD Patients	Renal Diet	AST, ALT	Placebo Pill	Four tablets of Propolis (125 mg)	365	Parallel	Intervention 61.39 Control 61.5	Brazil	14 F/18 M	
Not reported	Patients with type II diabetes	Usual	TG, LDL HDL, TC AST, ALT	Placebo Capsule	Capsule (500 mg) twice daily	90	Parallel	Intervention 55.4 Control 54.86	Iran	61 F/33 M	
None	At least one of following altered parameters: Fasting glycemia, Lipids profile, Blood pressure or Diabetes mellitus	Usual	TG, LDL, HD, LTC AST, ALT	Placebo	15 Drops twice daily	90	Parallel	Intervention 48 Control 44.5	Chile	51F/16 M	
None	Patients with type II diabetes	Usual	AST, ALT	Placebo Wheat Flour Capsule	Capsule (500 mg) twice daily	56	Parallel	Intervention 30.14 Control 34.11	Iran	60 F and M	
None	Patients with type II diabetes	Usual	TG, LDL HDL, TC	Placebo Pill	Propolis three times per day (300 mg)	84	Parallel	Intervention 51.3 Control 56.07	Iran	28 F/29 M	
None	Patients with type II diabetes	Diabetic Diet	TG, LDL, HDL, TC	Placebo Tablet	Brazilian green Propolis tablet	56	Parallel	Intervention 63.7 Control 62.9	Japan	34 F/46 M	

**Table 2:** Study quality and risk of bias evaluation done using Cochrane collaboration’s tool

Selective reporting	Incomplete outcome data	Blinding of outcome assessment	Blinding of participants and personnel	Allocation concealment	Random sequence generation	Author, year (ref.)
+	+	?	+	?	+	
+	+	?	?	+	?	
+	+	?	+	?	+	
+	+	?	+	?	+	
+	+	?	+	?	+	
+	+	?	+	?	+	

**Meta-analysis**

**Effects of propolis on the blood lipids**

**TG**

**Figure 2:**
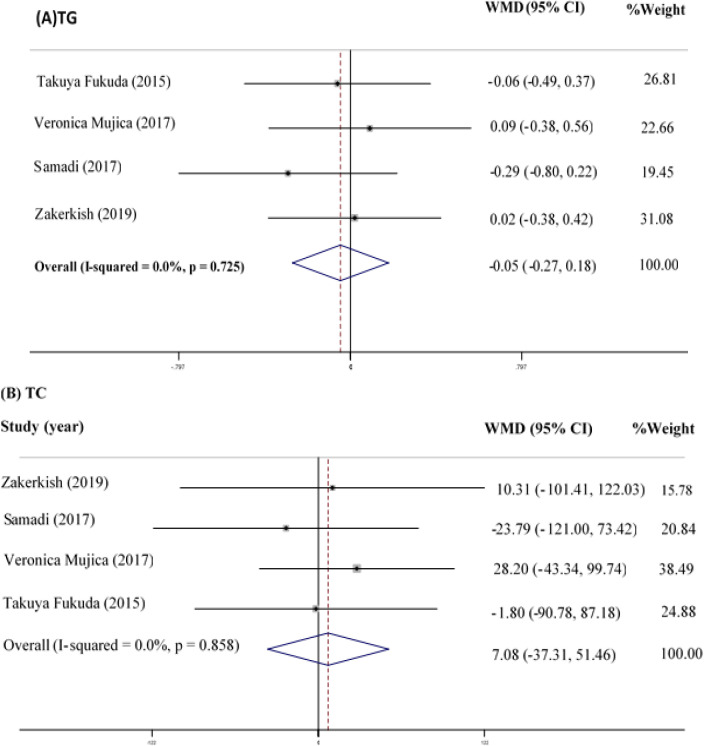
Forest Plot Detailing WMD and 95% CI for the impact of propolis supplementation on TG (A) and TC (B)

In total, four studies (Fukuda et al., 2015 ▶; Mujica et al., 2017 ▶; Samadi et al., 2017 ▶; Zakerkish et al., 2019 ▶) with 298 participants assessed the effects of propolis supplementation on TG as a favorable measurement. The overall analysis indicated a non-significant TG reduction in the subjects using propolis compared to the controls (WMD=-0.05 mg/dl; 95% CI: -0.27-0.18; p=0.688) (Figure 2A). Evidence of heterogeneity between the studies was not observed (Q statistic=1.32; I^2^=0.0%; p=0.725).

**TC**

The meta-analysis of the four RCTs (Fukuda et al., 2015 ▶; Mujica et al., 2017 ▶; Samadi et al., 2017 ▶; Zakerkish et al., 2019 ▶) indicated no significant difference in the TC values between the intervention and control groups (WMD=7.08 mg/dl; 95% CI: -37.31-51.46; p=0.755) (Figure 2B). In addition, evidence of heterogeneity between the studies was not observed (Q statistic=0.76; I^2^=0.0%; p=0.858). 

**LDL**

**Figure 3:**
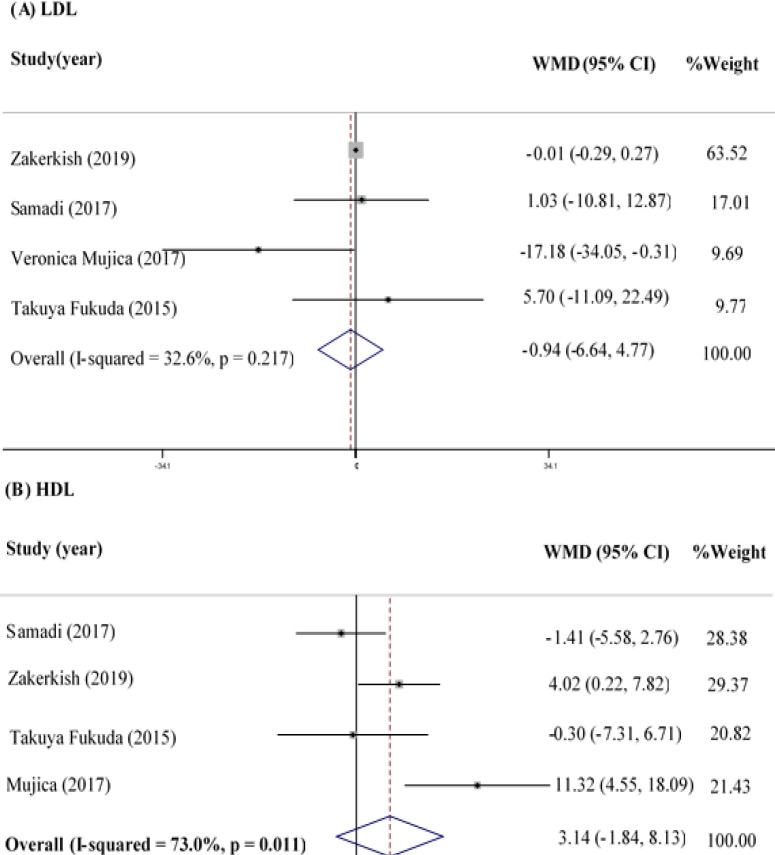
Forest Plot Detailing WMD and 95% CI for the impact of propolis supplementation on LDL (A) and HDL (B)

The effect of propolis supplementation on LDL was assessed in four clinical trials with 298 participants (Fukuda et al., 2015 ▶; Mujica et al., 2017 ▶; Samadi et al., 2017 ▶; Zakerkish et al., 2019 ▶). The meta-analysis 

showed that propolis supplementation could not change LDL significantly (WMD=-0.94 mg/dl; 95% CI: -6.64-4.77; p=0.747) (Figure 3A), and no heterogeneity was observed between the studies in this regard (Q statistic=4.45; I^2^=32.6%; p=0.217). 

**HDL**

In total, 298 participants were assessed in the four eligible studies (Fukuda et al., 2015 ▶; Mujica et al., 2017 ▶; Samadi et al., 2017 ▶; Zakerkish et al., 2019 ▶) in terms of HDL values, and no significant difference was detected in HDL between the study groups (WMD=3.14 mg/dl; 95% CI: -1.84-8.13; p=0.216) (Figure 3B). However, a significant heterogeneity was observed between the studies regarding the effects of propolis on HDL (Q statistic=11.13; I^2^=73.0%; p=0.011).

**Effects of propolis on liver enzymes**

**AST**

**Figure 4:**
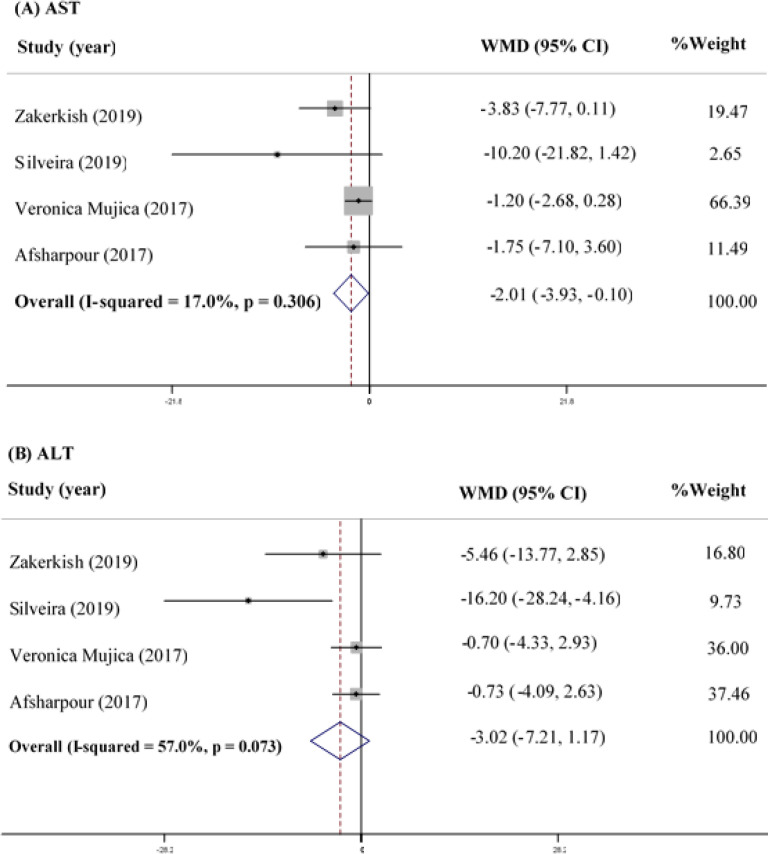
Forest Plot Detailing WMD and 95% CI for the impact of propolis supplementation on AST (A) and (B) ALT

Four studies on 253 participants assessed the effect of propolis supplementation on AST as a favorable measurement, and the analysis indicated that propolis supplementation significantly reduced the circulating AST levels (WMD=-2.01; 95% CI: -3.93--0.10; p=0.039) (Figure 4A). However, no heterogeneity was observed between the studies in this regard (Q statistic=3.61; I^2^=17.0%; p=0.306). 

**ALT**

According to the meta-analysis of the four RCTs (253 participants) regarding the data on ALT changes, propolis supplementation has been observed to reduce ALT compared to the controls, while the observed effect has not been considered significant (WMD=-3.02; 95% CI: -7.21-1.17; p=0.158) (Figure 4B). In addition, no heterogeneity was observed between the studies in this regard (Q statistic=6.97; I^2^=57.0%; p=0.073).

**Publication bias**

There was no evidence of publication bias in studies included in the meta-analyses as assessed by the asymmetry tests, except for the effect of propolis on serum ALT; the Egger’s regression test indicated that publication bias exists (p=0.042). So, the magnitude of publication bias was Asseyed with using trim & fill analysis.

## Discussion

As far as we know, this was the first meta-analysis to investigate the effects of propolis supplementation on the lipid profile and liver biomarkers, and the results do not support a positive impact for propolis supplementation on the lipid profile. Concerning the liver biomarkers, our findings confirmed the beneficial effects of propolis supplementation only on AST rather than the other liver biomarkers.

According to the present study, the participants administered

with propolis showed no significant reduction in TG, TC, LDL-c and ALT, and no significant increase was observed in HDL-c. Surprisingly, the AST levels were reported to decrease in the participants using propolis.

The results of this systematic review and meta-analysis are in accordance with the results of another study, demonstrating that supplementation with 226 mg/day of propolis for eight weeks had no significant effects on the components of the lipid profile (Fukuda et al., 2015 ▶). Inconsistently, the study by Samadi et al. indicated that 12 weeks of high-dose propolis supplementation (900 mg/day) resulted in better glycemic control, as well as slight TC and LDL-c increases (Samadi et al., 2017 ▶). Furthermore, several animal studies have shown that propolis supplementation could significantly reduce TC and the other components of the lipid profile (Fuliang et al., 2005 ▶; Attia et al., 2014 ▶; Kitamura et al., 2013 ▶). 

Although our findings did not confirm considerable effects for propolis supplementation on the lipid profile, it has been reported that propolis could modulate lipid metabolism (Fuliang et al., 2005 ▶). A possible mechanism is that the flavonoids in propolis may decrease the biosynthesis of cholesterol through inhibiting the hepatic 3-hydroxy-3-methylglutaryl-CoA reductase and acyl CoA: cholesterol o-acyltransferase (ACAT) (Bok et al., 1999 ▶). On the other hand, decreased ACAT activity may reduce the availability of cholesterol ester for very-low-density-lipoprotein cholesterol (VLDL-c) packing, which in turn reduces the secretion of VLDL-c from the liver (Carr et al., 1992 ▶; Kurowska and Manthey, 2004 ▶). Another possible mechanism is that propolis could reduce the activity of phosphatidylcholine-specific phospholipase C and increase the levels of annexin A7 in ox-LDL-stimulated endothelial cells and becomes involved in the modulation of dyslipidemia as well (Xuan et al., 2014 ▶).

Although our findings did not confirm positive effect for propolis on HDL-c, some studies have demonstrated that propolis supplementation could increase the levels of HDL-c and improve cardiovascular diseases (Mujica et al., 2017 ▶; Zakerkish et al., 2019 ▶; Azab et al., 2015 ▶). The positive effect of propolis on HDL-c could be attributed to the stimulation of pre-β HDL-C (Barakat and Mahmoud, 2011 ▶; Daniel, 2006 ▶). Propolis increases the expression of the liver ATP-binding cassette transporters A1 and G1 (ABCA1 and ABCG1) protein, which leads to cholesterol discharge from the peripheral tissue. Therefore, it could be concluded that propolis may be involved in the formation of HDL particles and could increase HDL-c levels (Nader et al., 2010 ▶; Yu et al., 2011 ▶).

The hepatoprotective effects of propolis have been well documented *in-vitro* and *in-vivo* (Kismet et al., 2008 ▶; Bhadauria et al., 2007 ▶; Paulino et al., 2014 ▶; Omar et al., 2016 ▶; Wali et al., 2015 ▶). In this systematic review and meta-analysis, we observed that propolis treatment significantly decreased AST levels, while it had no significant effects on the other liver biomarkers. The results of a recent study demonstrated that supplementation with Iranian propolis could significantly reduce the levels of the liver transaminases (ALT and AST) (Zakerkish et al., 2019 ▶). Another study reported that the administration of caffeic acid phenethyl ester, as an active component of propolis, exerted hepatoprotective effects by reducing the levels of the hepatic transaminases in diabetic rats (Tolba et al., 2013 ▶). Inconsistently, the study by Silveira et al. indicated that supplementation with green propolis had no beneficial effects on the liver transaminases (Silveira et al., 2019 ▶). The hepatoprotective effects of propolis may be related to its anti-inflammatory and antioxidant features as the oxidative stress and inflammatory cytokines that are generated by excessive accumulation of fat in the hepatocytes may conduce to neutrophil infiltration and cause inflammatory liver damage (Nabavi et al., 2015 ▶).

According to experimental animal liver damage models, propolis administration could improve the activity of the hepatic antioxidant enzymes, such as glutathione peroxidase, superoxide dismutase, and catalase (Kismet et al., 2008 ▶; Nakamura et al., 2010 ▶; Won Seo et al., 2003 ▶). However, propolis contributes to restoration of energy provision, thereby preventing lipoapoptosis, which is a major cause of lipotoxic liver injury and nonalcoholic steatohepatitis (Xiao et al., 2015 ▶; Jin et al., 2017 ▶). 

Another possible mechanism for the hepatoprotective effects of propolis is that propolis is associated with down-regulation of the expressions of well-known SREBP-1 responsive lipogenic genes, *FANS*, *SCD1*, and *FABP5*, which are effective in the prevention of lipid accumulation in the liver by reducing lipid synthesis and increasing the rate of fatty acid oxidation, which leads to decreased liver steatosis (Hulver et al., 2005 ▶; Listenberger et al., 2003 ▶; Ye et al., 2019 ▶). 

Notably, a wide spectrum of hepatoprotective effects have been ascribed to various flavonoids in propolis, such as pinocembrin, naringin, chrysin, and galangin (Ye et al., 2019 ▶). For instance, it has been suggested that naringin could operate as a protective and therapeutic factor in liver fibrosis through inhibition of reactive oxygen species generation, suppression of PI3K/Akt signaling-mediated cell survival, up-regulation of anti-inflammatory cytokines, and down-regulation of the profibrotic cytokines (El-Mihi et al., 2017 ▶). However, further trials are needed to explore the effects of propolis on the hepatic biomarkers.

**Strengths and limitations**

This was the first meta-analysis to comprehensively examine the effects of propolis based on the available RCTs regarding the lipid profile and liver biomarkers. The current meta-analysis was performed based on a systematic search to find all the related published literature regardless of whether the components of the lipid profile and liver biomarkers were the initial or secondary outcome. Therefore, publication bias is not expected in the analyses. Additionally, our analyses were limited to the RCTs that were based on the methodological criteria in order to diminish the potential biases. Most of the reviewed RCTs were double-blind, which enhances the inference of the cause-and-effect relationship. 

The study had several limitations; for instance, the review included only a small number of studies, and we were not able to perform subgroup analysis to assess the effects of study durations and type of populations on the changes in the lipid profile and liver biomarkers after propolis supplementation. Another limitation of the meta-analysis was the high inter-study heterogeneity, which might have affected the meta-analysis results.

In the current systematic review and meta-analysis, we demonstrated that propolis supplementation can be effective in reducing AST; nevertheless, there was no significant effect on lipid profile indices and ALT.
